# Smoking: a leading factor for the death of chronic respiratory diseases derived from Global Burden of Disease Study 2019

**DOI:** 10.1186/s12890-022-01944-w

**Published:** 2022-04-20

**Authors:** Hui Gan, Xiangqing Hou, Zheng Zhu, Mingshan Xue, Teng Zhang, Zhifeng Huang, Zhangkai Jason Cheng, Baoqing Sun

**Affiliations:** 1 National Center for Respiratory Medicine, The First Affiliated Hospital of Guangzhou Medical University, National Clinical Research Center for Respiratory Disease, State Key Laboratory of Respiratory Disease, Guangzhou Institute of Respiratory Health, Guangzhou, 510120 China; 2 Faculty of Health Sciences, University of Macau, Macau, 999078 China; 3 Department of Allergy and Clinical Immunology, Guangzhou Institute of Respiratory Health, State Key Laboratory of Respiratory Disease, The First Affiliated Hospital of Guangzhou Medical University, Guangzhou, 510120 China; 4 Guangzhou Eighth People’s Hospital, Guangzhou Medical University, Guangzhou, 510060 China

**Keywords:** Chronic respiratory disease, Global disease burden, Smoking, Particulate matter pollution

## Abstract

**Background:**

Smoking is believed as one of the major risk factors resulting in a variety of non-communicable diseases, such as lung cancer and chronic respiratory diseases (CRDs). However, the global burden of CRDs attributed to smoking has not been systematically studied, particularly across different temporal and spatial scales.

**Methods:**

We conducted a systematic analysis of the Global Burden of CRDs and related risk factors using data from the Global Burden of Disease Study 2019. Incidence, death, risk factors, and other parameters such as estimated annual percentage change have been analyzed. We also compared various risk factors across regions, countries, and genders.

**Results:**

Globally, the incidence of CRDs and deaths cases have increased in the last 30 years, while the corresponding age-standardized incidence rate (ASIR) and death rate (ASDR) have declined. Smoking was the leading risk factor for the death of CRDs all over the world. However, in low and low-middle Socio-demographic Index (SDI) areas, particulate matter pollution was the main risk factor leading to death from CRDs, while smoking was ranked first among the major risk factors in areas with middle, middle-high, or high SDI. Globally, gender differences in morbidity and mortality from CRDs were observed. Males had slightly more cases and ASIR of chronic respiratory diseases than females over the last 30 years. However, the mortality cases and ASDR in males were significantly higher than that of females. Furthermore, the ASDR of all major risk factors, specially smoking, was higher in men than in women.

**Conclusions:**

CRDs were still major threats human health. The current study highlights the dominating roles of smoking for death risks resulting from CRDs, followed by PM pollution. Therefore, tobacco control and improving air quality are key to reducing deaths from CRDs.

**Supplementary Information:**

The online version contains supplementary material available at 10.1186/s12890-022-01944-w.

## Introduction

Chronic non-communicable diseases (CNCDs) are responsible for the lion’s share of global death and disability, accounting for around 60% of all deaths worldwide. These diseases including chronic respiratory diseases (CRDs), cardiovascular diseases, cancers, and type 2 diabetes affect people of all ages, nationalities and classes [1, 2]. CNCDs have a far-reaching influence on populations that goes beyond illness and mortality, with significant financial consequences [3]. However, most CNCDs are preventable and curable, and it is effective to take necessary precautions against risk factors [4, 5]. In 2016, tobacco smoking was the leading risk factor causing CNCDs globally, followed by air pollution. In many areas, including Southeast Asia, air pollution is by far the leading cause of CNCDs [6]. Non-smoking policies, high-quality diet, and physical activity are one of the most effective elements in primary and secondary prevention of CNCDs [7].

Chronic respiratory diseases (CRDs), including chronic obstructive pulmonary disease (COPD), asthma, pneumoconiosis, interstitial lung disease (ILD) and sarcoidosis, and other diseases, all of which are CNCDs and important factors affecting human health [8]. Tobacco use, environmental pollution, occupational variables, and other risk factors have been shown to increase the incidence and mortality risks of CRDs [9]. Because CRDs, as one of the typical non-communicable diseases, was caused by a mix of genetic and environmental risk factors, identifying and prioritizing the primary risk factors for CADs would be beneficial for policy-marker when allocating medical and health resources. The global death toll from CRDs in 2017 was 3.91 (3.79–4.04) million, 1.18 times higher than in 1990 [10]. Although previous researchers have reported a worldwide overview of the global burden of CRDs study and their risk factors according to the GBD 2017 data [9], to the best of our knowledge, the epidemiological characteristics and dominant risk factors for CRDs deaths have not been well documented on a global scale based on the newly updated GBD 2019.

To fill this knowledge gap, this study aimed: (1) to systematically describe the incidence, deaths, age-standardized incidence rate (ASIR), and age-standardized death rate (ASDR) as well as trends burden of CRDs in 204 countries and regions based on GBD 2019; (2) to explore and prioritize the leading risk factors for death from CRDs at global, regional and country levels; and (3) to reveal regions, countries and gender differences of CRDs in terms of incidence, deaths, ASIR, and ASDR.

## Methods

### Study data

Data for the disease burden and risk factors of CRDs were derived from an online data source tool, the Global Health Data Exchange (GHDx) query tool (http://ghdx.healthdata.org/gbd-results-tool), which is an ongoing global collaboration that uses all available epidemiological data to provide a comparative assessment of health loss from 369 diseases across 204 countries and territories [11].From GBD 2019, we acquired data on the incidence, death, ASIR, ASDR, risk factors and estimated annual percentage change (EAPC) of CRDs from 1990 to 2019. The Socio-demographic Index (SDI), which is based on national-level income per capita, average years of education among people over the age of 15, and total fertility rate, was used to categorize the countries into five SDI quintiles (high, high-middle, middle, low-middle, and low levels). Details about the study design and methods of GBD studies have been extensively described in existing GBD literature [11–16].

### Data analysis

A secondary descriptive analysis of the burdens of CRDs in 204 countries and territories between 1990 and 2019 was carried out, and the findings were further investigated in different SDI regions. The number of incidence cases, deaths, risk factors, ASIR, and ASDR (per 100,000 population) in both genders were also compared. Bayesian meta-regression with DisMod-MR 2.1 was used as the primary method to estimate each condition. A generalized linear model was used to compute the EAPC of ASIR and ASDR among the risk factors. Uncertainty intervals (UIs) were defined as the 2.5^th^ and 97.5^th^ values of the posterior distributions. All code is freely available at GHDx.

All data management and statistical analyses were performed using SAS (version 9.4) and the figures were created using GraphPad Prism (version 9) or SAS (version 9.4).

## Results

### Incidence cases and deaths from CRD increased globally, but ASIR and ASDR declined

**Fig. 1:**
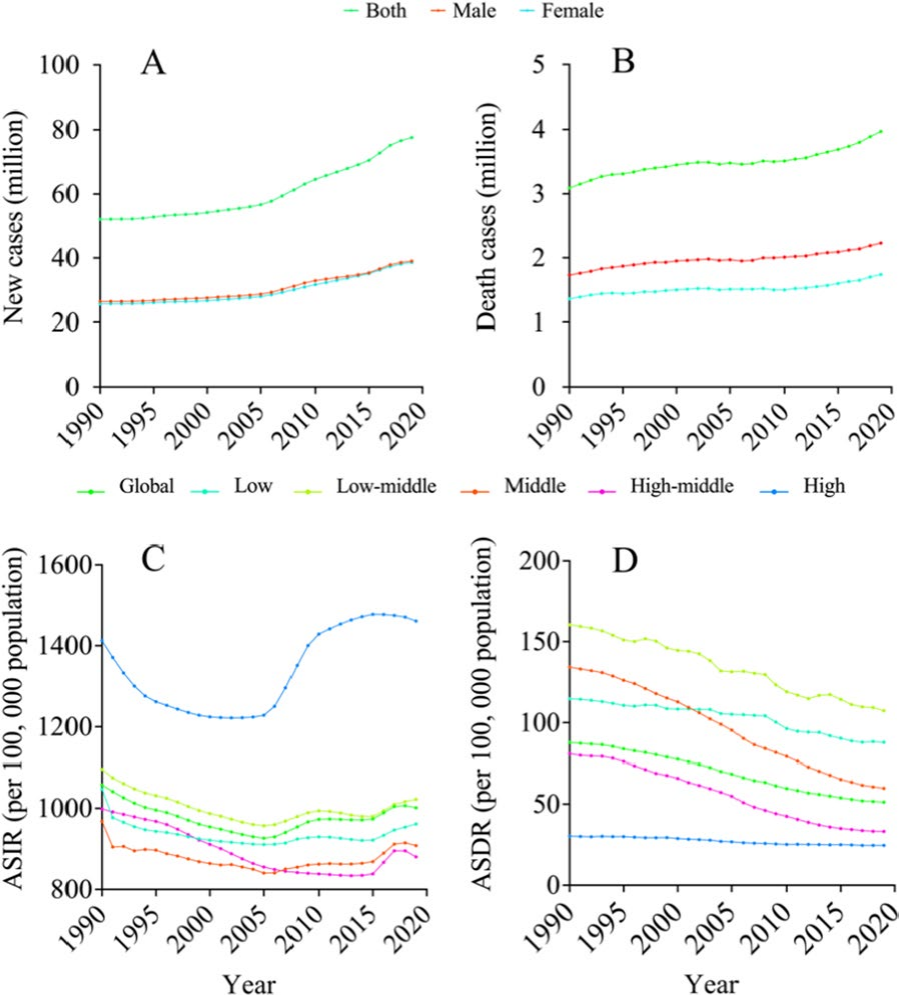
New cases (**a**), death cases (**b**), ASIR (**c**) and ASDR (**d**) of Chronic respiratory diseases globally and in different SDI regions from 1990 to 2019

From 1990 to 2019, the number of incidence cases and deaths of CRDs worldwide showed an increasing trend (Fig. 1a, b), greatly outnumbering new deaths. The number of new incidence cases in males was marginally greater than in females, but the number of deaths in males was significantly higher than in females (Fig. 1a, b).

In 1990, the global number of new cases of CRDs in the world was 52.09 million (95% UI: 45.47–60.63). It was 77.63 million (95% UI: 68.88–87.93). In 2019, an increase of 48.9% compared with 1990 (Table 1). In 1990, the global death toll from CRDs was 3.09 million (95% UI: 2.58–3.34); by the end of 2019, the figure had risen to 3.97 million (95% UI: 3.58–4.30), a 28.5% increase. Gender and socio-economic status had a great impact on the number of new cases and deaths of CRDs. In 1990 and 2019, the number of deaths from CRDs was significantly higher in males than that in females (Table 1). The number of new cases in the middle SDI region was the highest in both years. However, while the middle SDI regions had the highest number of deaths in 1990, the low middle SDI regions had the highest number of CRD deaths in 2019.

**Table 1:** Incidence cases and death cases of CRDs in 1990 and 2019

Characteristics	1990		2019	
	Incidence, new cases, million, 95% UI	Number of deaths, million, 95% UI	Incidence cases, million, 95% UI	Number of deaths, million, 95% UI
Global	52.09 (45.47–60.63)	3.09 (2.58–3.34)	77.63 (68.88–87.93)	3.97 (3.58–4.30)
*Gender*				
Male	26.41 (22.82–30.89)	1.73 (1.51–1.88)	39.01 (34.42–44.61)	2.23 (2.03–2.45)
Female	25.68 (22.65–29.74)	1.36 (1.02–1.51)	38.61 (34.36–43.39)	1.74 (.46–1.96)
*Sociodemographic index*				
Low	5.05 (4.22–6.09)	0.22 (0.19–0.25)	9.35 (7.84–11.23)	0.36 (0.31–0.40)
Low-middle	9.80 (8.52–11.32)	0.76 (0.63–0.85)	15.68 (13.91–17.72)	1.22 (1.03–1.38)
Middle	14.52 (12.17–17.36)	1.04 (0.82–1.15)	20.95 (18.45–23.96)	1.20 (1.06–1.34)
High-middle	11.01 (9.76–12.70)	0.74 (0.60–0.81)	13.35 (11.94–4.96)	0.65 (0.58–0.77)
High	11.69 (10.41–13.34)	0.32 (0.30–0.35)	16.63 (14.87–18.54)	0.53 (0.45–0.56)

In 1990, the ASIR of CRDs worldwide was 1057.45 (95% UI: 942.35–1202.87) per 100,000 population, but it was reduced to 1001.57 (882.99–1144.44) per 100,000 population in 2019, a 5.57% decrease from 1990 with an EAPC in ASIR of − 0.05 (95% UI: − 0.07 to − 0.04) (Additional file 1: Table S1).

Globally, the ASIR of CRDs was slightly higher in males in both 1990 and 2019 (Additional file 1: Table S1). In 1990, the ASIR of CRDs was 1096.81 (95% UI: 974.43–1246.84) per 100,000 population in male and 1027.37 (95% UI: 915.07–1166.39) per 100,000 population in females.

In 2019, the ASIR of CRDs was 1034.12(95% UI: 910.04–1187.41) per 100,000 population in males, and 973.44 (95% UI: 860.64–1108.64) per 100,000 population in females (Additional file 1: Table S1). The EAPC of ASIR of CRDs in males from 1990 to 2019 was slightly lower than that in females (male: − 0.06 (95% UI: − 0.08 to − 0.04), female: − 0.05 (95% UI: − 0.07 to − 0.04)), indicating that the incidence rate dropped slightly quicker in males than in females during the past 30 years.

For different SDI regions, the ASIR of CRDs in high SDI areas was the highest in 1990 and 2019. Furthermore, high SDI areas were the only ones with positive EAPC in all regions, with an incidence rate of 1412.44 (95% UI: 1231.53–656.49) per 100,000 population in 1990, and 1460.92 (95% UI: 1263.85–686.87) per 100,000 population in 2019. The ASIR of CRDs in the high SDI area has increased in the past 30 years, while the corresponding incidence rate was decreasing in other areas. In 1990, the middle SDI area had the lowest ASIR of CRDs, at 968.05 (95% UI: 851.44–1111.93) per 100,000 population, while the high-middle SDI area has the lowest incidence rate in 2019, at 880.44 (95% UI: 759.84–1029.04) per 100,000 population. Moreover, the middle SDI regions witnessed the fastest decline in ASIR over the past 30 years, with an EAPC of − 0.12 (95% UI: − 0.15 to − 0.09) (Additional file 1: Table S1).

Age-standardized death rates (ASDR) from CRDs have declined globally over the past 30 years (Fig. 1d). In 1990, the global ASDR for CRDs was 87.89 (95% UI: 73.87–95.10) per 100,000 population, compared to 51.28 (95% UI: 45.90–55.51) per 100,000 population in 2019. In 2019, it decreased by 41.65% compared with 1990, and the EAPC was − 0.42 (95% UI: − 0.48 to − 0.32) (Additional file 1: Table S1).

Globally, the ASDR of CRDs in males was much higher than that in females in both 1990 and 2019 (Additional file 1: Table S1). In 1990, the ASDR of CRDs of females was 67.84 (95% UI: 51.59–75.41) per 100,000 population, and that of males was 116.75 (95% UI: 102.61–126.76) per 100,000 population in the same period, which was 1.72 times of females. In 2019, the ASDR of CRDs of females was 39.73 (95% UI: 33.24–44.75) per 100,000 population, 66.72 (95% UI: 60.55–73.06) per 100,000 population for males, and 1.68 times that of females. ASDRs of CRDs were lower in 2019 than in 1990 for both males and females. In the past 30 years, the ASDR of CRDs in males has declined slightly quicker than in females (Additional file 1: Table S1).

In terms of trends, the ASIR of CRDs has fluctuated globally from 1990 to 2019, particularly in areas with high SDI (Fig. 1c). In the beginning, it decreased gradually before 2000, and then experienced a stable plateau period until 2005, when it increased significantly from 2005 to 2016 and then decreased slightly thereafter. However, the ASIR of CRDs in other regions exhibited an overall downward trend with some minor fluctuations. Prior to 2006, the middle SDI regions had the lowest incidence rate of CRDs, however after 2006, the lowest area changed to high-middle SDI regions. Surprisingly, the ASIR of CRDs was significantly higher in high SDI regions over the past 30 years than any in any other area (Fig. 1c).

From 1990 to 2019, the ASDR of CRDs showed a downward trend globally, which was directly tied to the economic and social levels. In high SDI regions, the ASDR of CRDs was the lowest and changed little over the past 30 years. On the contrary, the ASDR of CRDs in low-middle SDI regions has been at the highest level in all regions. (Fig. 1d). In 2019, the ASDR of CRDs in low-middle SDI regions reached the highest with 160.43 (95% UI:135.86–179.49) per 100,000 population, which was 5.3 times greater than that in high SDI regions, showing a huge difference (Additional file 1: Table S1).

In 2019, the ASDR of CRDs in Nepal is the highest among all the 204 countries and territories in the world, reaching 231.2 (95% UI: 175.79–270.35) per 100,000 population. The lowest was in Montenegro, 9.32 (95% UI: 7.48–10.91) per 100,000 population (Additional file 1: Figure S1-A). In the past 30 years, although the overall ASDR of CRDs declined globally, 140 countries and territories showed an upward trend. Bhutan, Nepal and Timor-Leste increased most rapidly (Additional file 1: Figure S1-B). It is particularly noteworthy that Nepal is not only the highest in ASDR but also at the forefront of the rising rate. This demonstrated that CRDs in Nepal not only had a high mortality rate, but they were also increasing rapidly, which calls for more attention and effort.

### ILD and sarcoidosis was the only kind of CRDs with increasing ASIR and ASDR

**Fig. 2:**
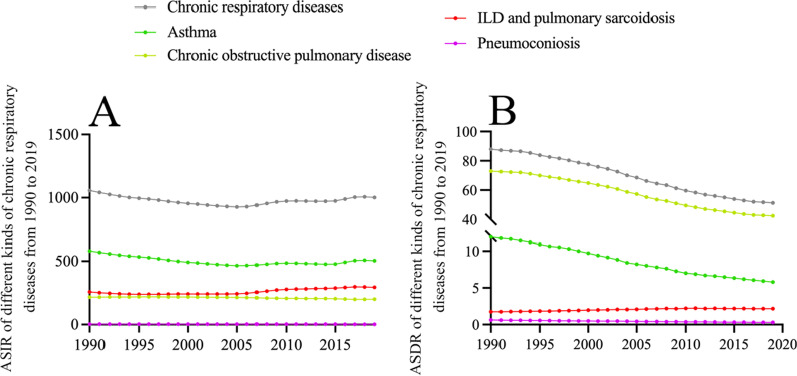
Age-standardized incidence and death rate (per 100,000) of four kinds of CRDs from 1990 to 2019 globally. **a** Age-standardized incidence rate (per 100,000); **b** Age-standardized death rate (per 100,000)

CRDs include asthma, COPD, pneumoconiosis, ILD and sarcoidosis. In the data of GBD 2019, interstitial lung disease and sarcoidosis are classified as a class of CRDs. In the past 30 years, the ASIR of CRDs showed a small fluctuation (Fig. 2a).

Asthma had the greatest ASIR of the four CRD categories from 1990 to 2019, but it showed a sluggish decline overall with a modest increase after 2016. Other CRDs with ASIRs ranging from high to low included ILD, sarcoidosis, COPD, and pneumoconiosis. The incidence of ILD and sarcoidosis has increased slightly over the past 30 years, but COPD and pneumoconiosis have stayed relatively stable and fluctuated less (Fig. 2a).

Globally, the ASDR from CRDs showed a gradual decline from 1990 to 2019 (Fig. 2b). COPD had the highest age-standardized death rate of any CRD, followed by asthma, ILD and sarcoidosis, and pneumoconiosis. In terms of changing trends, the ASDR of COPD, asthma, and pneumoconiosis have all shown a downward trend over the past 30 years, with COPD and asthma declining at a substantially faster rate. Only ILD and sarcoidosis had an increasing age-standardized death rate.

### Smoking is the leading risk factor for death from CRDs worldwide

**Fig. 3:**
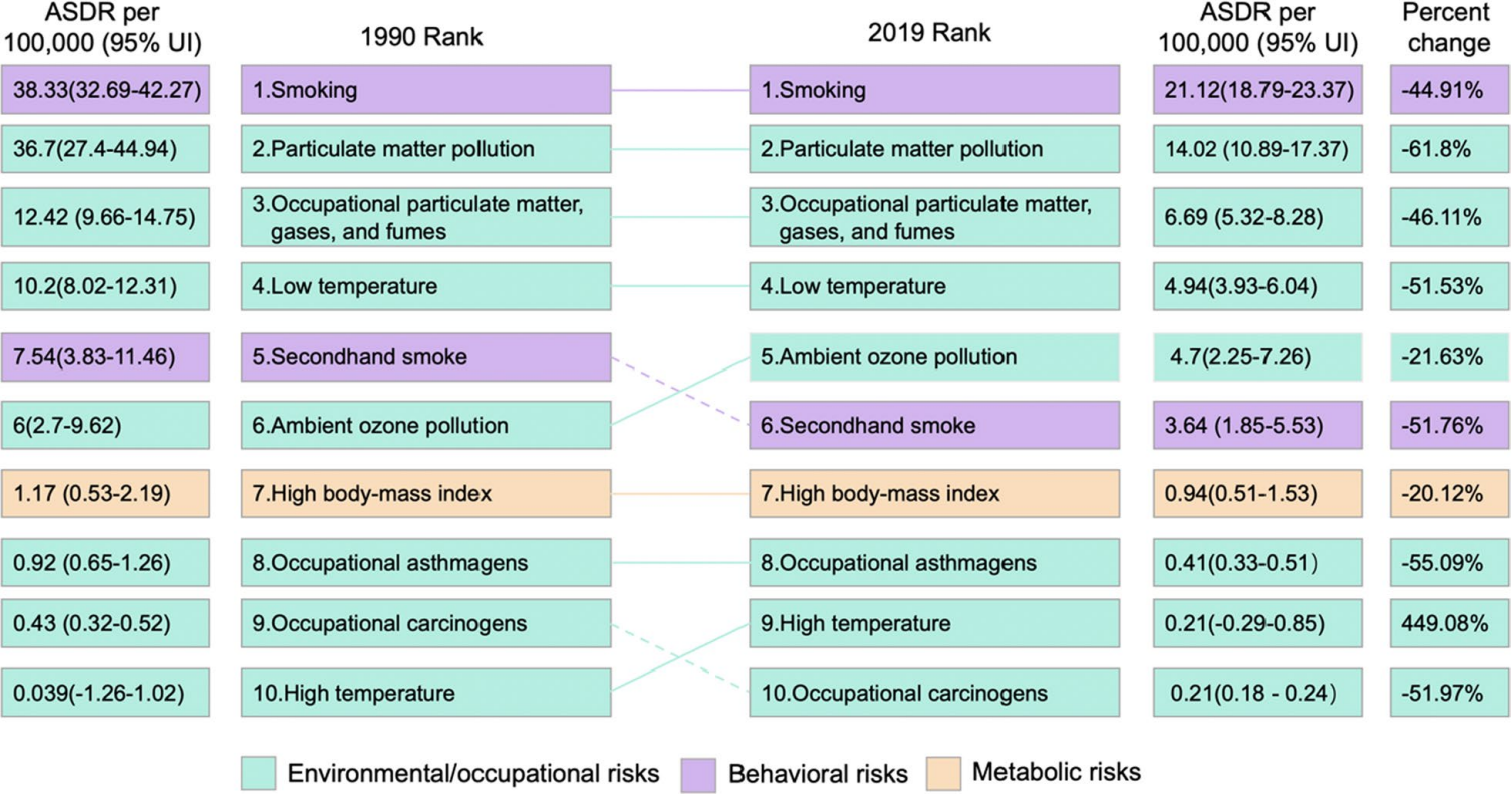
Comparison of the rankings and percentage changes in ASDR attributable to 10 risk factors in 1990 and 2019

GBD 2019 data indicated that smoking was the leading risk factor for the death of CRDs in 1990 and 2019, which was a typical behavioral factor. Followed by particulate matter (PM) pollution, occupational PM, gases, and fumes, and low temperature, all of which were environmental and occupational risk factors (Fig. 3). The other six risk factors are ambient ozone pollution, secondhand smoke, high body-mass index, occupational asthmagens, high temperature and occupational carcinogens. These risk factors have a relatively small impact on the death of CRDs, but they are also worthy of attention.

The ranking of the top four risk factors did not change both in 1990 and 2019. In 1990, the fifth largest risk factor was secondhand smoke, while in 2019 it was ambient ozone pollution. In 1990, the ASDR of CRDs caused by smoking was 1.04 times, 3.09 times and 3.76 times than that of PM pollution, occupational PM, gases, and fumes, and low temperature, respectively. In 2019, the death from CRDs caused by smoking was more prominent than that in 1990 (Fig. 3).

Among the top 10 major risk factors, only high temperature showed an increasing trend in terms of percent change of ASDR, from 0.039 (95% UI: − 1.26%–1.02) in 1990 to 0.21 (95% UI: − 0.29–0.85) per 100,000 population in 2019, an increase of 449.08%. The other nine major risk factors all showed a downward trend. The impact of PM pollution dropped the most compared to 1990, with a decrease of 61.8%; and the lowest was the high body-mass index, with a decrease of 20.12%. The most significant risk factor, smoking, has decreased by 44.91% since 1990 (Fig. 3).

### Risk factors for death from CRDs related to SDI levels with large differences between countries and regions

**Fig. 4:**
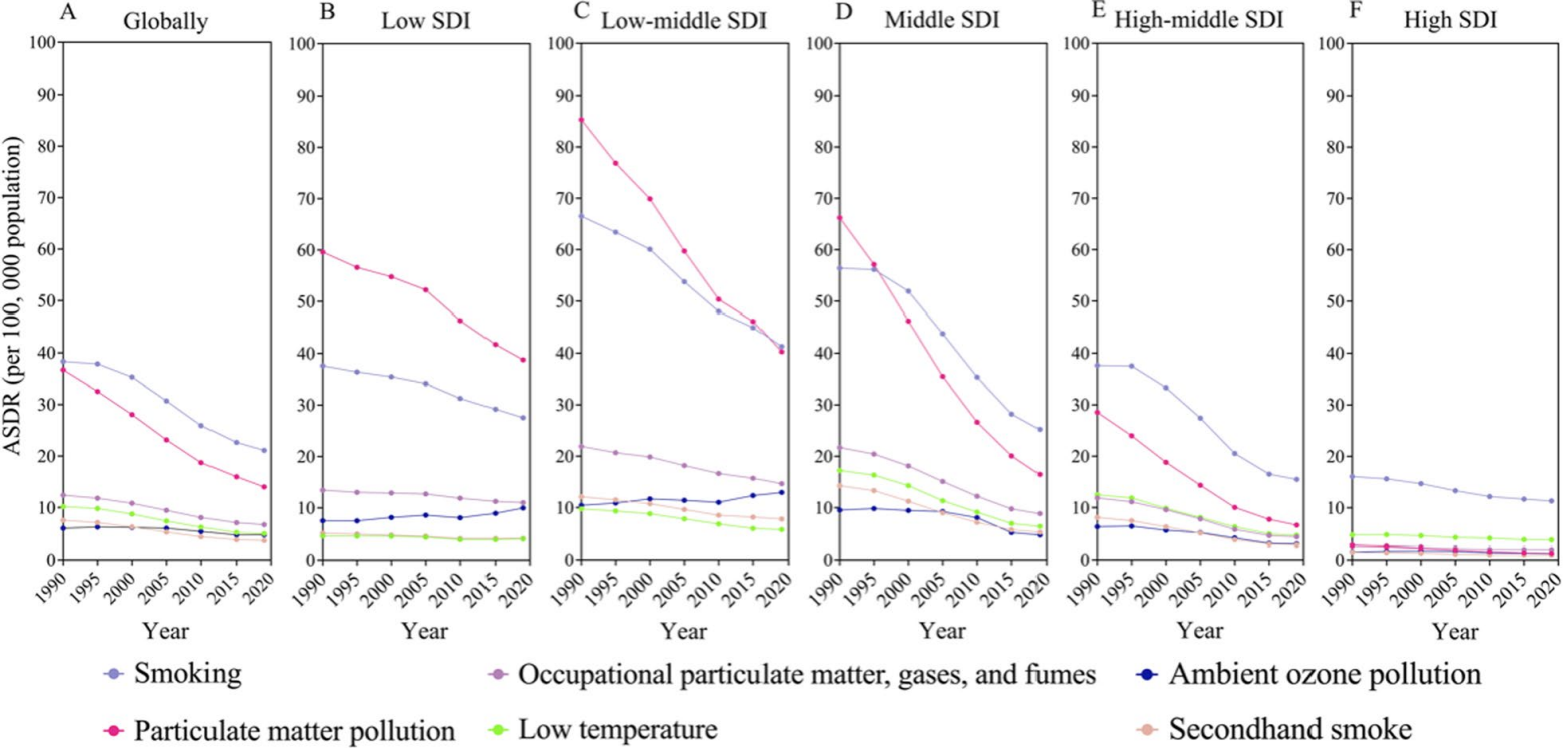
The ASDRs attributable to the top 6 risk factors by SDI region from 1990 to 2019. **a** Globally; **b** low SDI region; **c** low-middle SDI region; **d** middle SDI region; **e** high-middle SDI region; **f** high SDI region

We further analyzed the trends of six major risk factors in different SDI regions from 1990 to 2019. Globally, smoking was the leading risk factor for ASDR from CRDs, followed by PM pollution. Although both risk factors were declining, smoking fell more slowly than PM pollution. As a result, the ASDR gap between smoking and PM pollution has increased globally over the past 30 years. Significant disparities in major risk factors were found between different SDI regions. In low SDI areas, the biggest risk factor for ASDR from CRDs was PM pollution, which was substantially greater than smoking. Smoking, on the other hand, was the leading risk factor in both high-middle and high SDI areas. In addition, in areas with low-middle and middle SDI, the biggest risk factor has shifted over the last 30 years. Prior to 2017, the greatest risk factor in low-middle SDI areas was PM pollution, followed by smoking. After 2017, smoking became the first risk factor, and PM pollution dropped to the second. In the middle SDI areas, the biggest risk factor was PM pollution before 1995; and after that, it was smoking. With the exception of low SDI areas, ASDR from CRDs induced by PM pollution, smoking and occupational particle matter, gases, and fumes tend to decline with the improvement of SDI (Fig. 4). In low and low-middle SDI regions, ASDR from CRDs caused by ambient ozone pollution showed a slightly increase over the past 30 years, while five other risk factors declined. This shows the importance of having reducing air pollution and creating a healthy environment for the population's health. All six risk factors, however, showed a downward trend in the middle, high-middle, and high SDI regions (Fig. 4d–f).

**Fig. 5:**
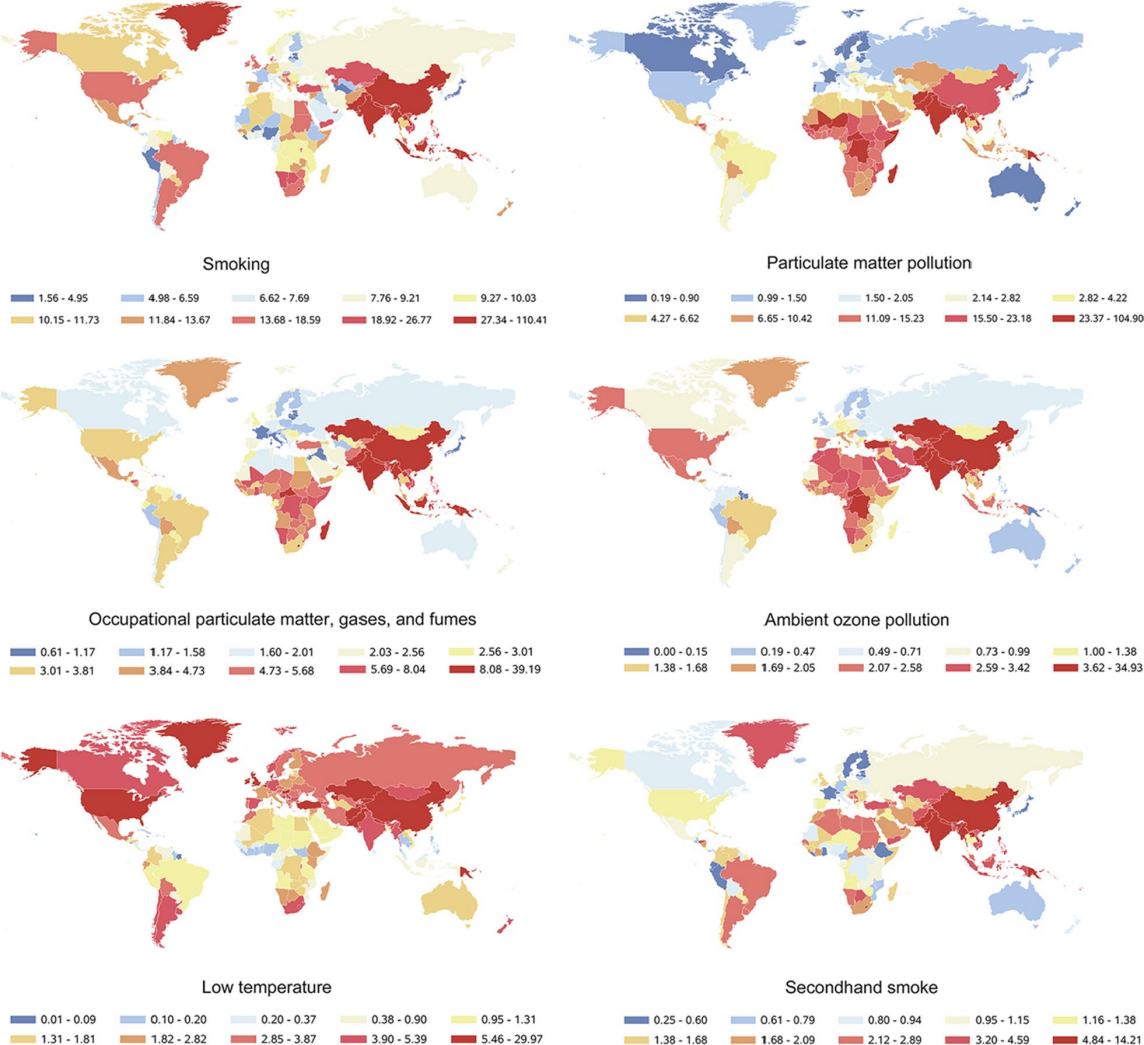
The ASDRs attributable to the top 6 risk factors in different countries and territories in 2019. Risk factors of ASDR of CRDs had gender differences related to SDI

The influence of six major risk factors on the ASDR of CRDs varies substantially across countries and territories in 2019. Of particular concern are the high levels of deaths rate from CRDs caused by all six risk factors in China and India, the world's two largest developing countries. Our findings showed that deaths from CRDs were prominent in China and India in 2019 (Fig. 5).

**Fig. 6:**
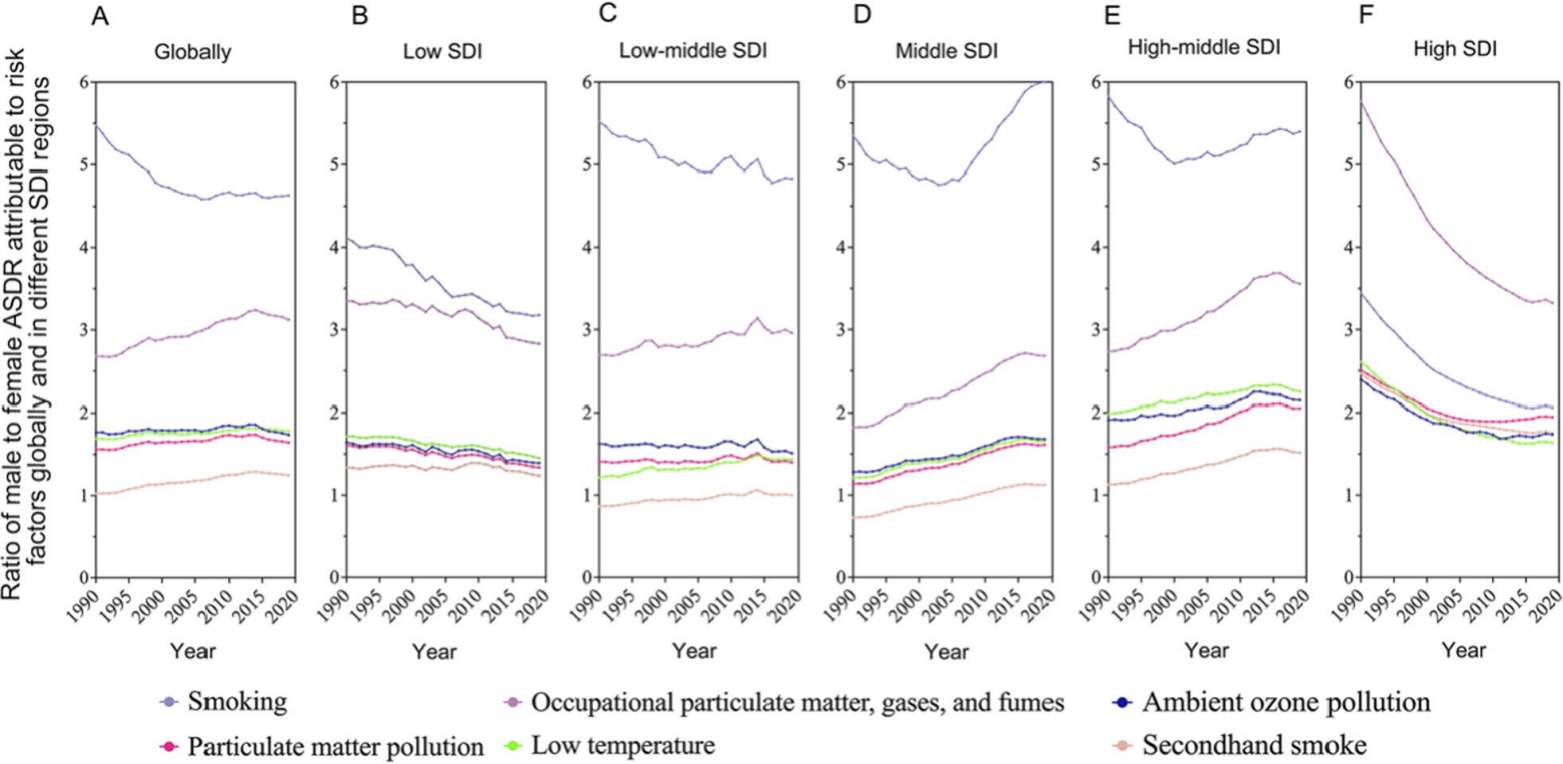
Ratio of male to female ASDR attributable to risk factors globally and in different SDI regions from 1990 to 2019. **a** Globally; **b** low SDI region; **c** low-middle SDI region; **d** middle SDI region; **e** high-middle SDI region; **f** high SDI region

Based on the previous analysis, we have shown that there were significant gender disparities in risk factors for ASDR of CRDs. We further analyzed gender differences by the ratio of ASDR of CRDs caused by the top six risk factors in male and female in different SDI regions. Globally, the ASDR of CRDs induced by the top six risk factors in male was higher than that in female from 1990 to 2019. Smoking was the most prominent, followed by occupational PM, gases, and fumes, ambient ozone pollution, and low temperature. The risk factor with the smallest gender gap is secondhand smoke. This indicated that smoking was a major source of the sex-dependent differences in CRDs fatalities from 1990 to 2019 (Fig. 6a).

In regions with different SDI levels, the ASDR of CRDs caused by various risk factors for males and females was quite varied (Fig. 6b–f). Except for the low-middle and middle SDI regions, the death rate caused by the secondhand smoke was higher in females than that of males. All of the other risk factors were higher in males than females. In high SDI regions, the risk factor with the greatest difference between males and females was occupational PM, gases, and fumes (Fig. 6f). In other regions, smoking was the biggest difference (Fig. 6b–e). For different SDI regions, the ratio of death caused by various risk factors for males and females varied substantially from 1990 to 2019, especially smoking and occupational PM, gases, and fumes.

## Discussion

GBD 2019 contains data on disease burden and risk factors of 369 diseases in 204 countries from 1990 to 2019 [11]. Based on these data of GBD 2019, we described and analyzed the disease burden of CRDs, especially focusing on their risk factors. Totally, the incidence and mortality of CRDs from 1990 to 2019 increased globally while the corresponding ASIR and ASDR decreased. In subgroup analysis, the ASIR and ASDR of CRDs were larger in males than females, and the ASIR was higher in high SDI regions than other regions, while the lowest ASDR can be seen in high SDI regions among all regions during the past 30 years. Our findings, in particular, highlight the dominating roles of smoking in the risks of the global burden of CRDs, as well as the urgent need to manage the increasing burdens of CRDs caused by multiple risks factors in China and India areas. We believe that a thorough examination of risk factors will aid in the development of specific directions and strategies for the prevention and control of CRDs.

According to the GBD database 2019, CRDs mainly include four kinds of diseases: asthma, COPD, pneumoconiosis, ILD and sarcoidosis. These chronic noncommunicable diseases have a huge impact on human health. Despite the fact that the number of CRD cases and fatalities have increased dramatically over the last 30 years, the number of new deaths is significantly less than the number of new cases, thanks to effective treatment and management approaches. Additionally, our results suggest that the number of deaths of CRDs in males was significantly higher than that in females, but the number of new cases in males and females was similar from 1990 to 2019. We hypothesized that the vulnerability of males to CRDs-related deaths was mainly attributed to the fact that males are much sicker and respond poorly to treatment than females. In line with our findings, earlier studies also reported significant gender differences in airway diseases [17], and have shown that male patients with COPD were more likely to die following an acute exacerbation [18].

However, the age-standardized incidence and death rate of CRDs have been decreasing trend globally, whereas the incidence of cases and deaths has been growing. The most likely cause of the growing numbers of cases and deaths is mostly population growth and aging. Moreover, the ASIR of males was similar to that of females, but the death rate of males was significantly higher compared with females. Again, our results highlight that CRDs are more severe and likely to result in death in males, owing to the fact that male smokers far outnumbers female smokers [19], and smoking is the greatest risk factor leading to death from CRDs worldwide.

The morbidity and mortality rates of CRDs varied dramatically across SDI regions. The number of new cases of CRDs in middle SDI areas was the highest in 1990 and 2019. However, the highest incidence rate of CRDs in 2019 can be observed in the high SDI area, which was the only area with an increasing trend in the past 30 years. In 1990, the biggest number of deaths occurred in the middle SDI regions, and this shifted to low-middle SDI regions in 2019. Although the ASIR of CRDs in high SDI regions ranked first from 1990 to 2019, the corresponding ASDRs were the lowest. Our findings revealed that socioeconomic level promoted the incidence of CRDs, but high socio-economic level significantly reduced the probability of dying from CRDs. The imbalance of socio-economic levels leads to great differences in global disease mortality [20, 21].

From 1990 to 2019, the incidence rate of CRDs was relatively stable, with a slightly decreasing trend, but the ASDR showed a significant downward tendency since the treatment of CRDs has improved significantly in the past 30 years. More specifically, the asthma-induced ASIR was highest among CRDs, while the incidence rate was relatively stable. Additionally, the ASDR of asthma exhibited a significant downward trend as a result of the in-depth understanding of asthma, which encourages the advancement of asthma treatment and management. In particular, the use of glucocorticoids and the introduction of bio-targeted medicines in recent years have made asthma easier to control, lowering asthma mortality [22–26]. COPD had a lower ASIR during the past 30 years than asthma, but it had the highest death rate among four kinds of CRDs. Over the past 30 years, the ASDR of COPD has also shown a downward trend, but at a slower rate than that of asthma. COPD was the biggest burden of death in CRDs, and it may still be difficult to improve this situation in the future [27]. Among the four types of CRDs, ILD and sarcoidosis were the only kind of CRDs with an increase in age-standardized incidence and death rate. It is also the second-most common CRD after asthma.

The risk factors for ASDR of CRDs have not altered significantly in the past 30 years. Smoking is the leading risk factor of death from CRDs, followed by PM pollution. The ASDR of CRDs caused by these two risk factors was significantly higher than by other risk factors. Although the death rate caused by these two risk factors has been decreasing over the last 30 years, the decline rate of smoking has been slower than that of PM pollution, resulting in an expanding gap between the death rate caused by smoking and by PM pollution. In conclusion, smoking is expected to be the greatest risk factor of death caused by CRDs in the future. Therefore, tobacco control is the key to curbing the death caused by CRDs. Many nations have been adopting various methods to reduce tobacco consumption and have seen positive results, but more practical efforts are still required [19, 28]. About 25% of men and 5.4% of women were smokers globally, posing a significant barrier to controlling tobacco [28]. Smoking has an impact not only on the health of smokers, but secondhand smoking also has a greater impact on the health of others. In 2019, secondhand smoking is the sixth leading risk factor for death from CRDs. Furthermore, tobacco not only has a huge negative impact on CRDs, it also has great impacts on cardiovascular diseases, lung tumors and fertility [29–31].

In the past 30 years, the second leading risk factor for death from CRDs was PM pollution, but in 2019 it was down by 61.8% compared to 1990. At present, humans are paying increasing attention to environmental protection and the control of air pollution. It is believed that the impact of air pollution on CRDs will be further reduced in the future. Among the top 10 risk factors, the high temperature was the only risk factor that led to an increase in the ASDR of CRDs. Although its impact on CRDs was minor in comparison to other risk factors, it has increased by 449.08% in 2019 compared to 1990. The increase in global temperature caused by climate change has resulted in the deterioration of respiratory disorders, an increase in the incidence of asthma and COPD, as well as the incidence of allergies and respiratory infections [32, 33]. In fact, humanity has recognized the enormous economic, social, and health consequences of global climate change, but it still faces huge challenges and problems [34].

The impact of major risk factors on CRDs varies greatly between SDI regions. It has been suggested that in areas with high SDI levels, the death rate of CRDs caused by the risk factors was relatively small. On the contrary, the death rate of CRDs caused by the risk factors in low SDI areas was comparatively high. The level of SDI is directly associated with population health, and the huge disparity between the rich and the poor in many parts of the world has an impact on health equity [34, 35]. As the two largest developing economies, India and China, their top six risk factors had a relatively strong impact on the ASDR of CRDs. This may be related to the side effects of rapid economic expansion of the two nations, namely, environmental deterioration, air pollution, urbanization, and increased tobacco consumption. Governments should pay more attention to environmental protection, and implement stricter restrictions on tobacco consumption, especially in areas with the most negative effect.

## Conclusions

In general, although the age-standardized incidence and death rate of CRDs have been decreasing in the past 30 years, with the population expansion and population aging, the number of new cases and deaths of CRDs continues to climb. Moreover, there is obvious geographical heterogeneity in the global burden of CRDs, which was closely related to the level of SDI. It indicates that global health inequalities were prevalent. Smoking and PM pollution was the most prominent risk factors for death from CRDs. Therefore, controlling tobacco consumption and curbing environmental pollution were the key directions for reducing the burden of CRDs in the future.

## Supplementary Information

**Additional file 1: Table S1**. Age-standardized Incidence rates and Age-standardized Death rates due to chronic respiratory disease globally and different SDI regions in 1990 and 2019, and their estimated annual percentage changes (EAPC) from 1990 to 2019.**Figure S1**. ASDR (per 100,000) in 2019 (A) and EAPC of ASDR from 1990 to 2019 (B) of chronic respiratory disease in 204 countries and territories.

## Data Availability

The datasets analyzed during the current study are available in the IHME Data (http://ghdx.healthdata.org/gbd-results-tool). GBD 2019 data are free for non-commercial user and did not require any permissions to download. Public access to the GBD 2019 data is open. The raw data from GBD 2019 can be freely downloaded without any requirement for non-commercial users.

## Abbreviations

CRDs: Chronic respiratory diseases
ASIR: Age-standardized incidence rate
ASDR: Age-standardized death rate
SDI: Socio-demographic Index
CNCDs: Chronic non-communicable diseases
ILD: Interstitial lung disease
COPD: Chronic obstructive pulmonary disease
GHDx: Global Health Data Exchange
UIs: Uncertainty intervals
EAPC: Estimated annual percentage change
PM: Particulate matter
