# Supplementary material for: Smoking: a leading factor for the death of chronic respiratory diseases derived from Global Burden of Disease Study 2019
Source: BMC Pulm Med. 2022 Apr 20;22:149. doi: 10.1186/s12890-022-01944-w (PMC9019969; doi:10.1186/s12890-022-01944-w)

**Supplementary materials**

**Table S1:** Age-standardized Incidence rates and Age-standardized Death rates due to chronic respiratory disease globally and different SDI regions in 1990 and 2019, and their estimated annual percentage changes (EAPC) from 1990 to 2019

|  | ASIR per 100, 000 people, (95% UI) | | EAPC of ASIR,  %, 95% UI | ASDR per 100, 000 people, (95% UI) | | EAPC of ASDR,  %, 95% UI |
| --- | --- | --- | --- | --- | --- | --- |
|  | 1990 | 2019 |  | 1990 | 2019 |  |
| Global | 1057.45 (942.35-1202.87) | 1001.57 (882.99-1144.44) | -0.05 (-0.07 to -0.04) | 87.89 (73.87-95.10) | 51.28 (45.90-55.51) | -0.42 (-0.48 to -0.32) |
| Gender | | | | | | |
| Male | 1096.81 (974.43-1246.84) | 1034.12 (910.04-1187.41) | -0.06 (-0.08 to -0.04) | 116.75 (102.61-126.76) | 66.72 (60.55-73.06) | -0.43 (-0.50 to -0.34) |
| Female | 1027.37 (915.07-1166.39) | 973.44 (860.64-1108.64) | -0.05 (-0.07 to -0.04) | 67.84 (51.59-75.41) | 39.73 (33.24-44.75) | -0.41 (-0.50 to -0.26) |
| SDI regions | | | | | | |
| Low | 1047.71 (929.44-1184.30) | 961.70 (852.91-1094.95) | -0.08 (-0.10 to -0.07) | 114.69 (96.34-133.08) | 87.82 (74.17-97.67) | -0.23 (-0.34 to -0.14) |
| Low-middle | 1096.34 (977.34-1220.24) | 1022.38 (907.93-1141.98) | -0.07 (-0.08 to -0.05) | 160.43 (135.86-179.49) | 107.29 (90.10-120.82) | -0.33 (-0.44 to -0.24) |
| Middle | 968.05 (851.44-1111.93) | 908.19 (789.51-1050.90) | -0.06 (-0.08 to -0.04) | 134.26 (106.75-147.00) | 59.82 (52.30-66.61) | -0.55 (-0.61 to -0.42) |
| High-middle | 998.71 (881.44-1151.72) | 880.44 (759.84-1029.04) | -0.12 (-0.15 to -0.09) | 80.81 (64.52-87.92) | 33.22 (29.45-39.31) | -0.59 (-0.64 to -0.41) |
| High | 1412.44 (1231.53-656.49) | 1460.92 (1263.85-686.87) | 0.03 (0.00-0.07) | 30.30 (30.30-33.21) | 24.64 (21.49-26.07) | -0.19 (-0.28 to -0.15) |

**Figure S1.** ASDR (per 100,000) in 2019 (A) and EAPC of ASDR from 1990 to 2019 (B) of chronic respiratory disease in 204 countries and territories.


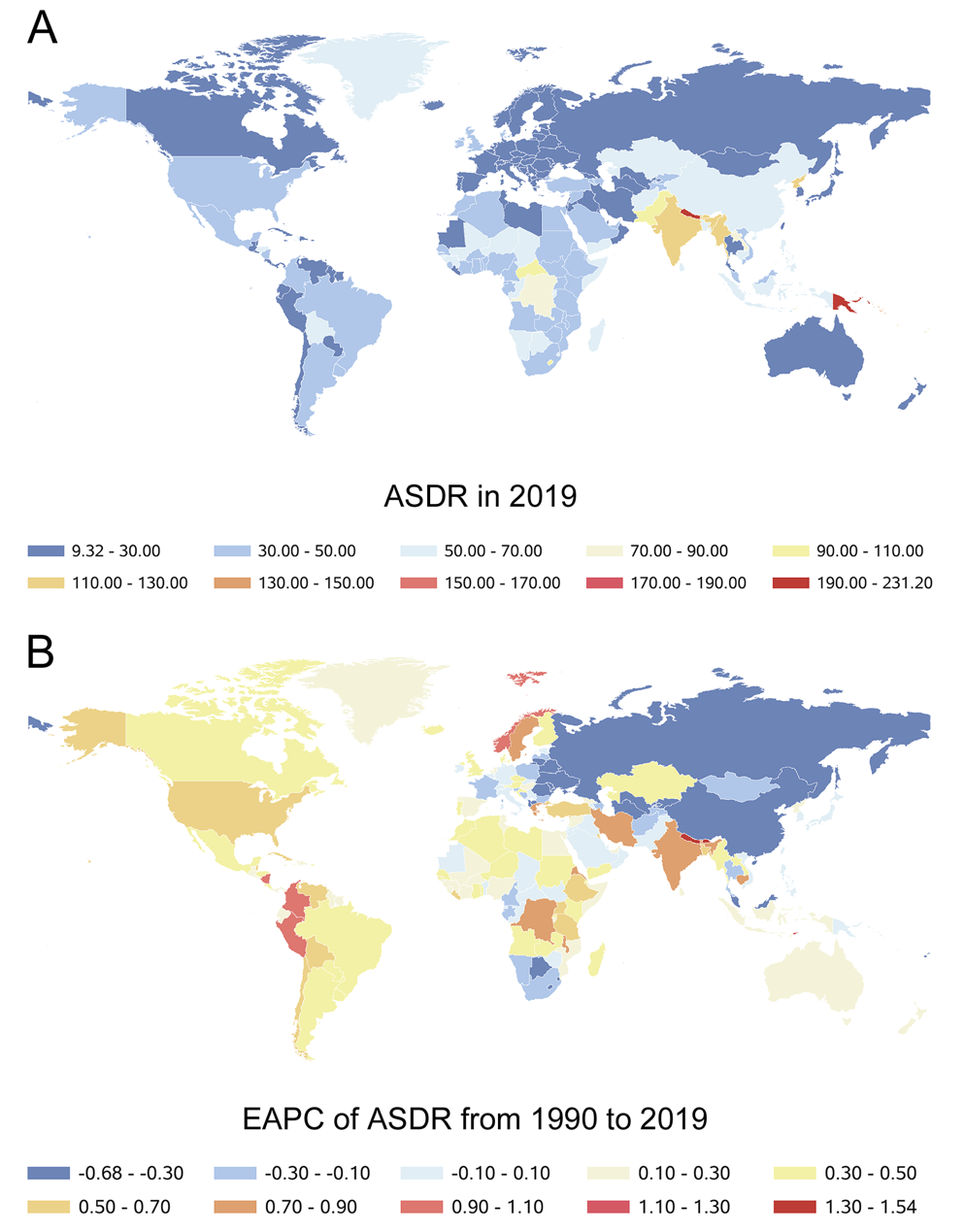

Supplement: Supplementary file 1 — Additional file 1: Table S1. Age-standardized Incidence rates and Age-standardized Death rates due to chronic respiratory disease globally and different SDI regions in 1990 and 2019, and their estimated annual percentage changes (EAPC) from 1990 to 2019.Figure S1. ASDR (per 100,000) in 2019 (A) and EAPC of ASDR from 1990 to 2019 (B) of chronic respiratory disease in 204 countries and territories. [file 12890_2022_1944_MOESM1_ESM.docx]
